# Fast Healthcare Interoperability Resources for Inpatient Deterioration Detection With Time-Series Vital Signs: Design and Implementation Study

**DOI:** 10.2196/42429

**Published:** 2022-10-13

**Authors:** Tzu-Wei Tseng, Chang-Fu Su, Feipei Lai

**Affiliations:** 1 Department of Computer Science and Information Engineering National Taiwan University Taipei City Taiwan; 2 Graduate Institute of Biomedical Electronics and Bioinformatics National Taiwan University Taipei City Taiwan

**Keywords:** Fast Healthcare Interoperability Resources, FHIR, Health Level 7, HL7, health research, data sharing, health information technology, clinical research

## Abstract

**Background:**

Vital signs have been widely adopted in in-hospital cardiac arrest (IHCA) assessment, which plays an important role in inpatient deterioration detection. As the number of early warning systems and artificial intelligence applications increases, health care information exchange and interoperability are becoming more complex and difficult. Although Health Level 7 Fast Healthcare Interoperability Resources (FHIR) have already developed a vital signs profile, it is not sufficient to support IHCA applications or machine learning–based models.

**Objective:**

In this paper, for IHCA instances with vital signs, we define a new implementation guide that includes data mapping, a system architecture, a workflow, and FHIR applications.

**Methods:**

We interviewed 10 experts regarding health care system integration and defined an implementation guide. We then developed the FHIR Extract Transform Load to map data to FHIR resources. We also integrated an early warning system and machine learning pipeline.

**Results:**

The study data set includes electronic health records of adult inpatients who visited the En-Chu-Kong hospital. Medical staff regularly measured these vital signs at least 2 to 3 times per day during the day, night, and early morning. We used pseudonymization to protect patient privacy. Then, we converted the vital signs to FHIR observations in the JSON format using the FHIR Extract Transform Load application. The measured vital signs include systolic blood pressure, diastolic blood pressure, heart rate, respiratory rate, and body temperature. According to clinical requirements, we also extracted the electronic health record information to the FHIR server. Finally, we integrated an early warning system and machine learning pipeline using the FHIR RESTful application programming interface.

**Conclusions:**

We successfully demonstrated a process that standardizes health care information for inpatient deterioration detection using vital signs. Based on the FHIR definition, we also provided an implementation guide that includes data mapping, an integration process, and IHCA assessment using vital signs. We also proposed a clarifying system architecture and possible workflows. Based on FHIR, we integrated the 3 different systems in 1 dashboard system, which can effectively solve the complexity of the system in the medical staff workflow.

## Introduction

### Background

Vital signs have been an important indicator in many studies [1-3]. In recent years, researchers have used these data in studies of predictive models for in-hospital cardiac arrest (IHCA) [1,4]. In a real-world medical workflow, complete data may be obtained once every 4 to 8 hours. In the machine learning research related to vital signs [5], the features include heart rate, temperature, respiratory rate, systolic blood pressure, and diastolic blood pressure. In addition to IHCA risk assessment, data analysis systems [6] and early warning systems [7] are still indispensable applications.

Although IHCA risk indicators have facilitated breakthroughs in machine learning [8,9], it has been difficult to integrate them into the workflow of medical staff. In hospitals, there are dozens of systems that must exchange information with each other. Without a standard exchange interface [10], the integration process is costly and time-consuming when a new application is imported. In addition, if medical researchers are allowed to access patient data directly through the health care information system database, security risks [11] become a concern.

To begin initiating a human-readable and user-friendly interface for medical staff, Health Level 7 [12] developed Fast Healthcare Interoperability Resources (FHIR) [13]. FHIR is a platform specification that defines a set of capabilities used across the health care processes, and it defines a generic health care business entity model that uses resources as the basic blocks. Each resource in FHIR has a defined relationship resource with data elements and constraints. In addition, the FHIR profile standardizes the data format and structure constraints. During data transportation, it uses the HTTP RESTful application programming interface (API) in the exchange interface and provides the flexibility to choose between JSON or XML format in the data payload.

### Aim

Although FHIR have developed some of the resources, a vital signs profile [14] has not yet matured. The current implementation guide provided by FHIR is insufficient to encompass the full range of medical system applications; therefore, hospitals still need to define the customized implementation guide to develop their system and workflow. The implementation guide is a collection of rules applied by FHIR resources [15] that requires a clear explanation of how to solve a particular problem. In the relevant studies on FHIR [16-18], each paper develops and discusses a single customized resource profile on a mobile device. Although FHIR can effectively and rapidly improve health care information system interoperability, it still has not proposed an implementation guide for the machine learning application in FHIR implementation guide registry. To accelerate the development of smart health care, we propose a system architecture process based on FHIR that can integrate the machine learning models. Besides, the vital signs applications are distributed in many different systems. This study can effectively solve the complexity of the system in the medical staff workflow.

To standardize the format among medical systems, we developed a complete IHCA implementation guide based on FHIR that defines the vital signs–related data for both the early warning system and the machine learning pipeline. In addition, we also developed FHIR Extract Transform Load (ETL) and other FHIR-related applications, including data management, an early warning system, and a machine learning pipeline.

## Methods

### Ethics Approval

This study was approved by the Institutional Review Board of the En-Chu-Kong Hospital (ECKIRB1071001). We confirm that all experiments were performed in accordance with relevant guidelines and regulations. The data retrieved from electronic health records (EHRs) were deidentified by an IT specialist and could not be linked to the patients’ identity by the research team. The need for written informed consent was waived and confirmed by the En-Chu-Kong Hospital Institutional Review Board, because this was a retrospective cohort study with deidentified data.

### Overview

Our study provides a design and implementation process for IHCA-based interoperability of health care information systems, and our design steps include use cases as well as the IHCA implementation guide.

In the use cases section, we describe the integration issues faced by health care institutions. Then, in the IHCA implementation guide section, we introduce the method used to migrate data from the healthcare information system (HIS) database to the FHIR server as well as a method for mapping the data to the FHIR resources. We also develop the 3 application systems, which include data management, early warning systems, and a machine learning pipeline. If used according to our implementation guide, the applications can easily obtain patient information and vital signs data.

### Use Case Survey

In health care institutions, the database is centrally managed, but the applications are developed by many different teams. In addition, medical staff usually access all of the required information about a workflow through a single system. Therefore, the interoperability of health care systems is very important.

To achieve system information interoperability [19], the HTTP RESTful API was defined to exchange data with other systems. However, many medical systems are legacy systems, and in many cases, it is impossible to change the system architecture. We therefore created a table view for the HIS database to allow other systems to obtain particular data. To avoid affecting the original system architecture, we developed FHIR ETL to convert data from the HIS database to the FHIR server, and FHIR ETL was implemented according to the rules defined by the IHCA implementation guide.

We interviewed 10 experts regarding health care system integration and information exchange. As shown in Table 1, FHIR, which has a good medical standard interface, is very suitable for solving the interoperability problems faced by medical information systems. In addition, it supports a variety of systems that can be used to develop extended applications.

**Figure 1:**
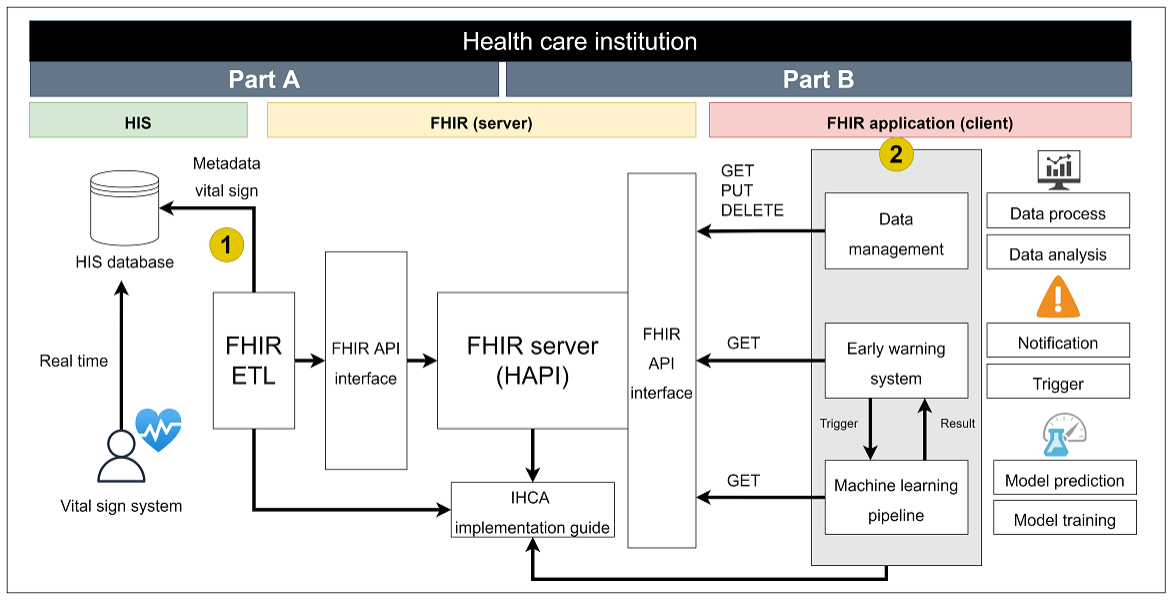
Use cases for IHCA research and application. (A) Extract the data and transfer them to the FHIR server. (B) Data management for data processing, early warning system for notification and model trigger, and machine learning pipeline for model prediction and model training. API: application programming interface; ETL: Extract Transform Load; FHIR: Fast Healthcare Interoperability Resources; HAPI: Health Level 7 application programming interface; HIS: healthcare information system; IHCA: in-hospital cardiac arrest.

Therefore, we have 2 use cases. The first use case is related to data migration for the FHIR server, as shown in Figure 1 (Part A). The second use case is related to FHIR applications, as shown in Figure 1 (Part B).

**Table 1:** Requirement list from health care specialists in health care institutions.

Issue (requirement)	How to do it?
The new system integration process shall not affect the health care information system or the vital signs system.	Build the FHIR^a^ server as a new middleware or gateway so that researchers can access data.
Converting the EHRs^b^ with vital signs into FHIR resources.	Develop the FHIR ETL^c^.
To reduce the time cost and compatibility, we need to use a health care information interoperability standard.	Use FHIR resources and the RESTful API^d^.
The field needs an early warning system that can continuously monitor the patient’s vital signs.	Use FHIR to develop the early warning system.
How can an organization integrate the prediction model into the medical workflow?	Use FHIR to develop the machine learning pipeline.
The field needs a complete implementation procedure and use case.	Define an FHIR implementation guide.

^a^FHIR: Fast Healthcare Interoperability Resources.

^b^EHRs: electronic health records.

^c^ETL: Extract Transform Load.

^d^API: application programming interface.

### IHCA Implementation Guide

In this phase, we need to consider the data format so that raw data can be transferred into FHIR resources as well as how the HTTP RESTful API can be used to easily obtain data. Therefore, we designed a system architecture (Figure 1). We divided the system steps into the following: (1) the FHIR ETL performs data conversion and comparisons between the HIS database and the FHIR server, and (2) the application system accesses data directly through the FHIR API interface at the HTTP layer.

### Data Mapping—FHIR ETL

We proposed the data mapping table to develop the FHIR ETL, as shown in Table 2. We defined the data mapping and resource relations. Based on the FHIR vital signs profile, we used the observation resource to store systolic blood pressure, diastolic blood pressure, heart rate, respiratory rate, and body temperature. According to Table 2, FHIR ETL can extract the data from the HIS database and transfer them to resource content.

**Table 2:** The data mapping table of the FHIR^a^ ETL^b^ in this study.

Data model of HIS^c^ database	FHIR resource name	FHIR resource attribute	Description
Patient_ID	Patient	identifier	An identifier for the patient in the hospital
Patient_name	Patient	name	Patient’s name that is human-readable
Gender	Patient	gender	Patient’s gender
BirthDate	Patient	birthDate	Patient’s birth date
Practitioner_ID	Practitioner	identifier	An identifier for the physician in the hospital
Practitioner_name	Practitioner	name	Physician’s name that is human-readable
Organization_ID	Organization	identifier	An identifier for the department in the hospital
Organization_name	Organization	name	Department’s name that is human-readable
Location_ID	Location	identifier	An identifier for the location in the hospital
Location_name	Location	name	Location’s name that is human-readable
Heart rate	Observation	valueQuantity.value	Heart rate
Temperature	Observation	valueQuantity.value	Temperature
Respiratory rate	Observation	valueQuantity.value	Respiratory rate
Systolic blood pressure	Observation	valueQuantity.value	Systolic blood pressure
Diastolic blood pressure	Observation	valueQuantity.value	Diastolic blood pressure
Timestamp	Observation	effectiveDateTime	The created time of the value

^a^FHIR: Fast Healthcare Interoperability Resources.

^b^ETL: Extract Transform Load.

^c^HIS: healthcare information system.

**Figure 2:**
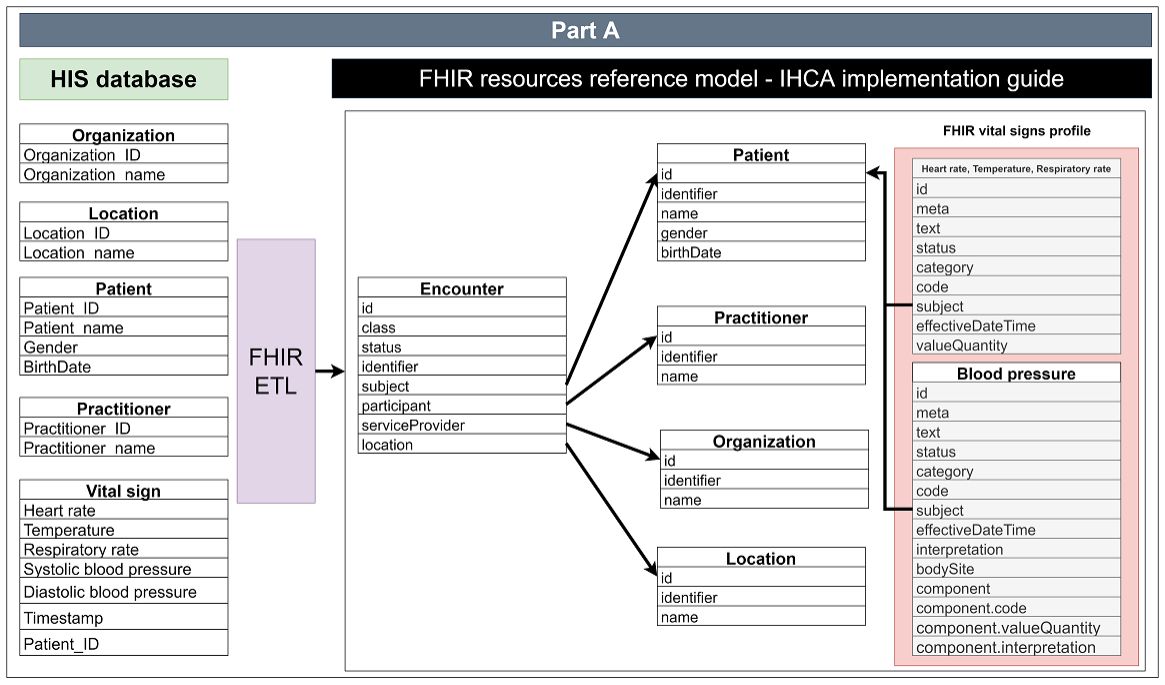
Data mapping and resource relationships in the IHCA implementation guide. ETL: Extract Transform Load; FHIR: Fast Healthcare Interoperability Resources; HIS: healthcare information system; IHCA: in-hospital cardiac arrest.

In Figure 2, in terms of data acquisition, if an FHIR client wants to obtain a patient’s location, it needs to first obtain the patient’s ID and join the encounter subject. Then, it can use the encounter location to find the location resource. Finally, the FHIR client can obtain the patient location.

**Figure 3:**
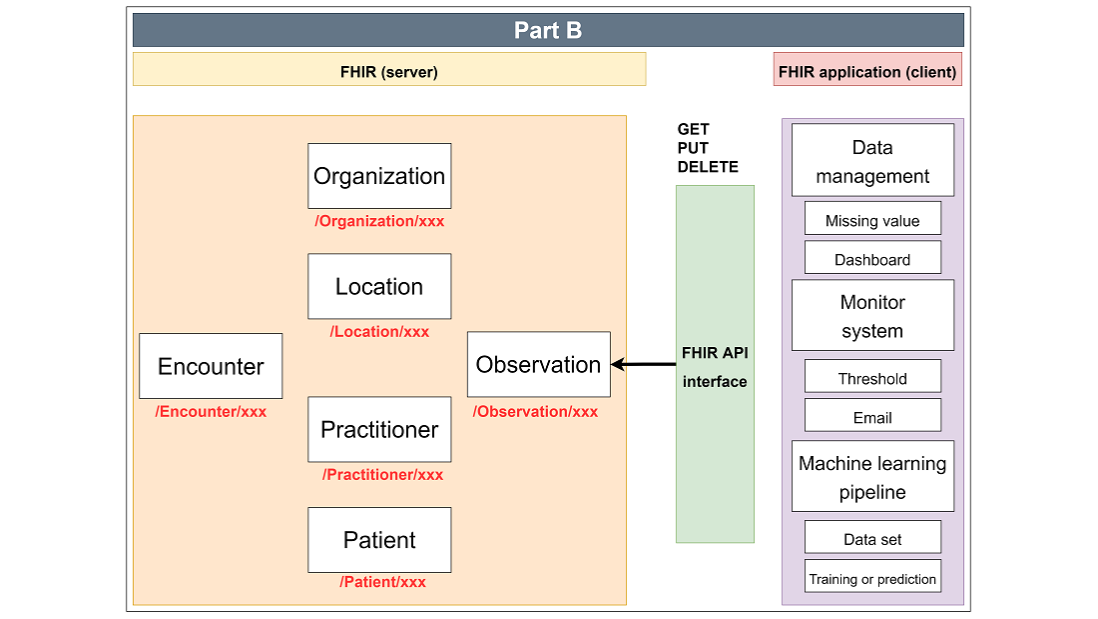
FHIR (Fast Healthcare Interoperability Resources) application, which uses the HTTP RESTful API (application programming interface) to control the data on the FHIR server.

In Figure 3, the FHIR client can perform the following: (1) when an FHIR client needs to access a particular patient using metadata, it can use the HTTP GET method to obtain the Bundle resource response; (2) when an FHIR client wants to update the location name for the hospital, it can use the HTTP PUT method to update the Location resource; and (3) after the FHIR client obtains sufficient vital signs data from the Observation resource, it can use the HTTP DELETE method to delete the resource that is missing vital signs values.

### Workflow Design

In this section, we describe the complete workflow of FHIR implementation. Workflow 1 develops the data mappings for the FHIR resources. First, the FHIR ETL uses the database connection library to access the table view of the HIS database. Then, it verifies that the patient’s information exists. To maintain data consistency, when converting to the Observation resource, the system must add the universally unique identifier of Patient resource as a reference link. If the patient’s basic data already exists, the vital signs will be converted into an Observation resource based on the FHIR vital signs profile.

**Figure 4:**
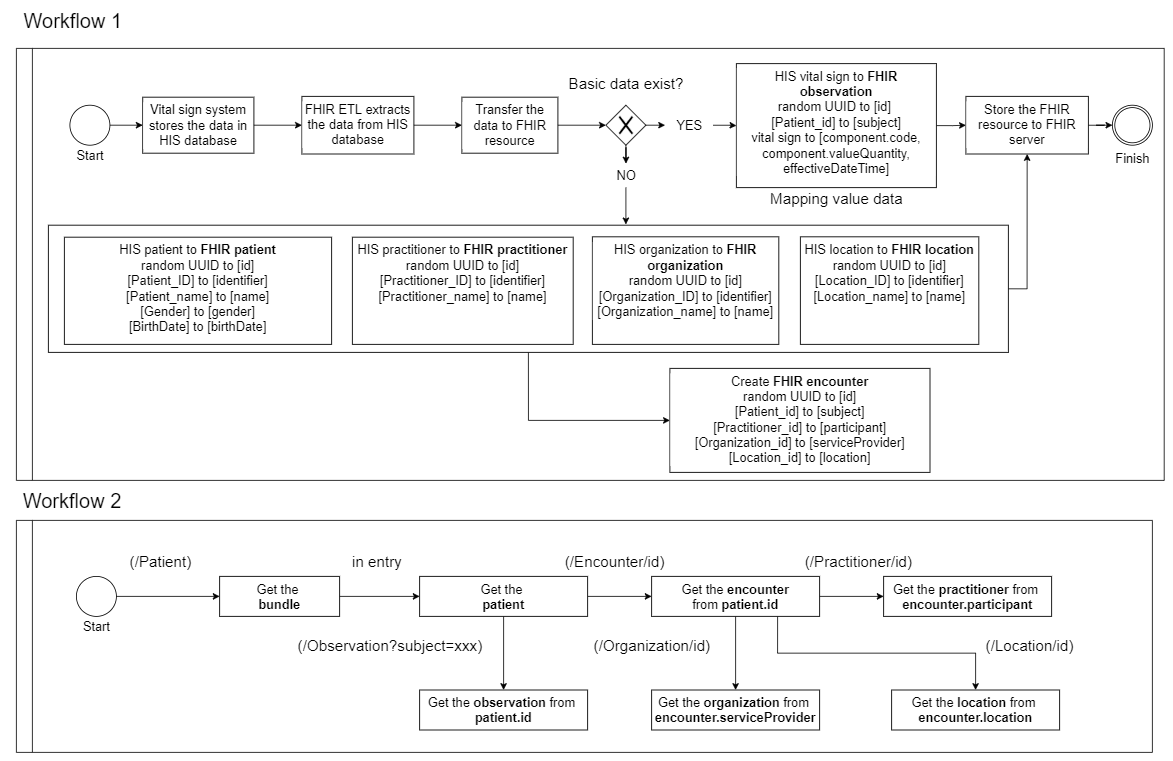
Workflow of the Fast Healthcare Interoperability Resources (FHIR) Extract Transform Load (ETL) and the FHIR client application. HIS: healthcare information system; UUID: universally unique identifier.

Workflow 2 develops the data acquisition process for FHIR applications. First, the FHIR application can use URL (/Patient) with the HTTP GET method to access the Bundle resource. In the Bundle resource, the FHIR application can find all of the patient’s data. If the FHIR application needs to obtain patient information such as location and practitioner information, it can use the Patient ID to join the Encounter subject. Then, it can obtain the Encounter participant and Encounter location. Finally, it can also use the Patient ID to join the URL (/Observation?subject=) with the HTTP GET method to obtain the Observation resource (Figure 4).

## Results

### FHIR Resources

The FHIR ETL is an automation service that extracts vital signs. When the vital signs system stores the data in the HIS database, the FHIR ETL can access the vital signs data immediately, and as shown in Figure 2, it adds the vital signs to the Observation resource. Multimedia Appendix 1 shows examples of an FHIR resource that refers to an FHIR vital signs profile and other resources.

### Software Development

**Figure 5:**
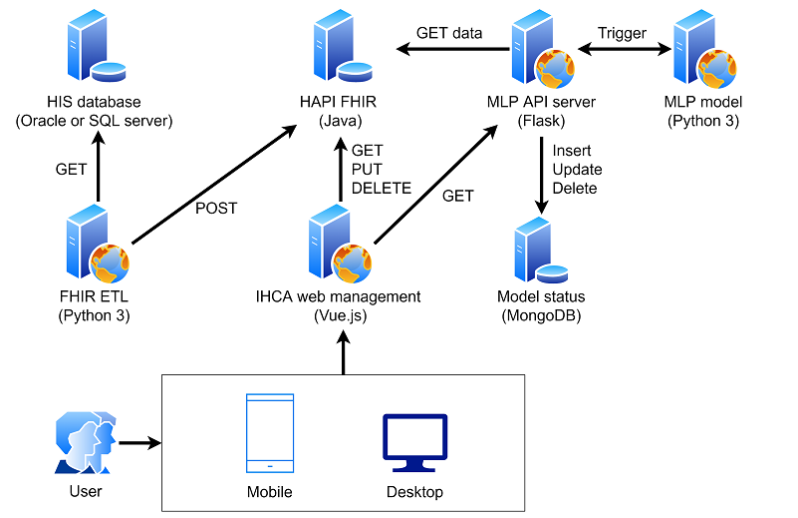
System architecture used in this study. API: application programming interface; ETL: Extract Transform Load; FHIR: Fast Healthcare Interoperability Resources; HAPI: Health Level 7 application programming interface; HIS: healthcare information system; IHCA: in-hospital cardiac arrest; MLP: machine learning pipeline.

We describe the software development, which is shown in Figure 5. The HIS database was developed using the SQL server database and the Oracle database server. The FHIR server was installed on the Health Level 7 API FHIR R4 server (version 6.1.0) [20] with a docker container based on the Java environment. This open-source system is widely used. We developed the back-end service of the FHIR ETL using Python software (version 3; Python Software Foundation), and the machine learning pipeline was implemented using Flask. The front-end website was constructed using Vue.js and is used for IHCA web management.

### System Implementation

The study data set [21] included the EHRs of adult inpatients who visited the En-Chu-Kong hospital. Medical staff regularly measured these vital signs at least 2 to 3 times per day during the day, night, and early morning. The total number of patients was 16,865, and the number of patients with IHCA was 118.

**Figure 6:**
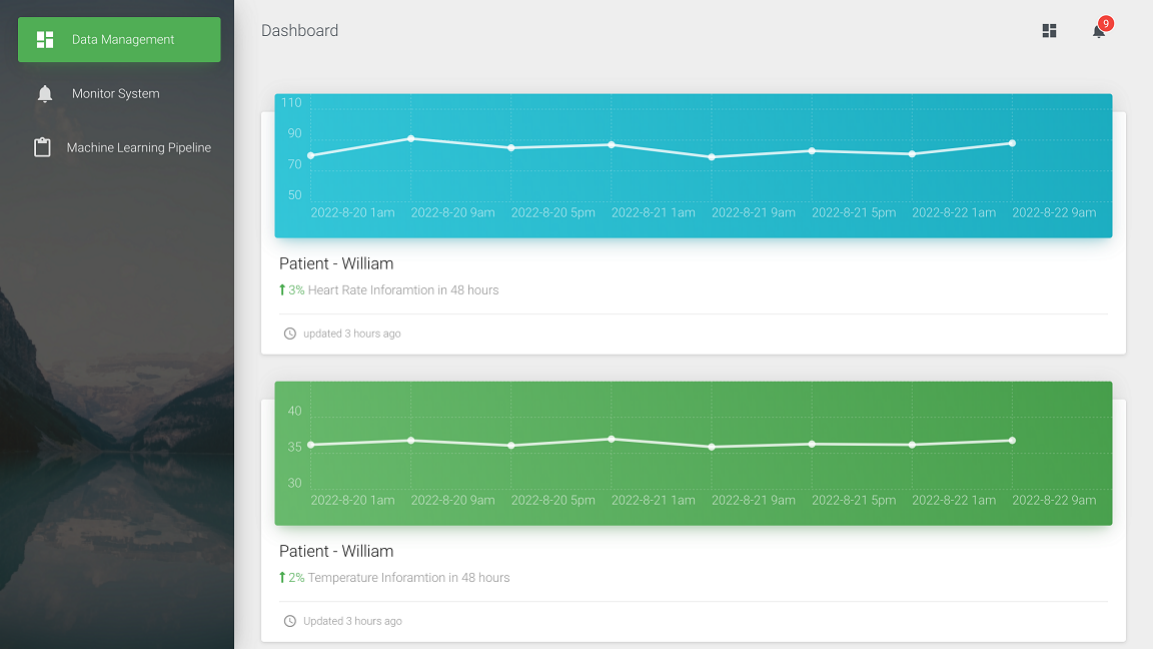
Screenshot of the data management overview in the dashboard.

**Figure 7:**
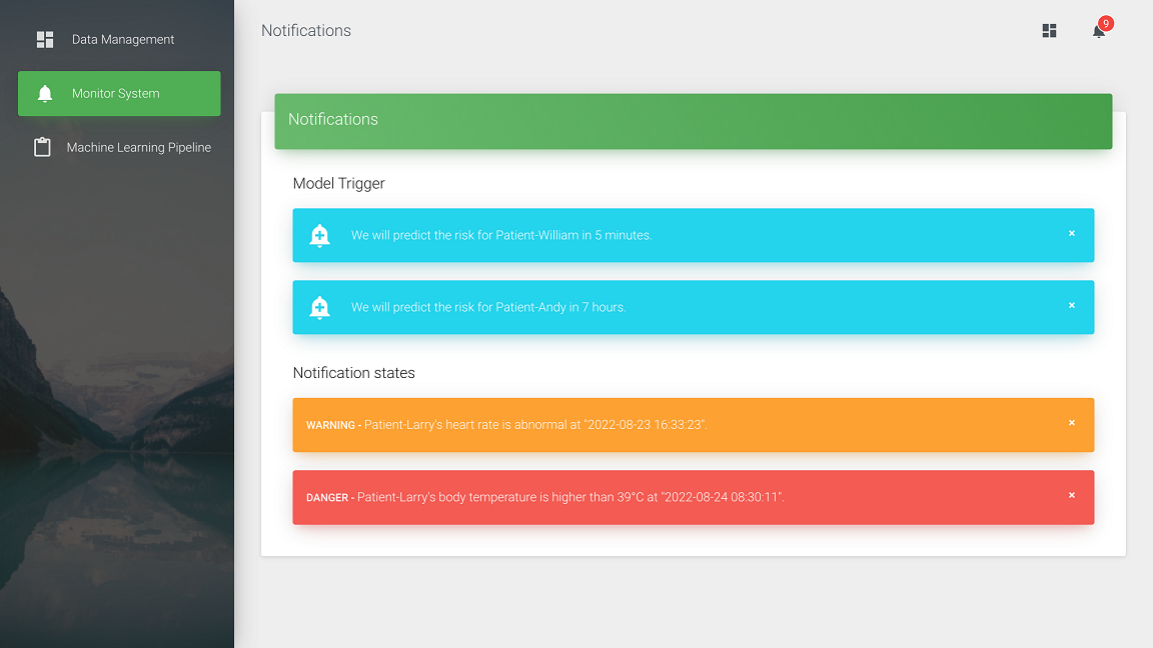
Screenshot of the early warning system’s notification overview.

**Figure 8:**
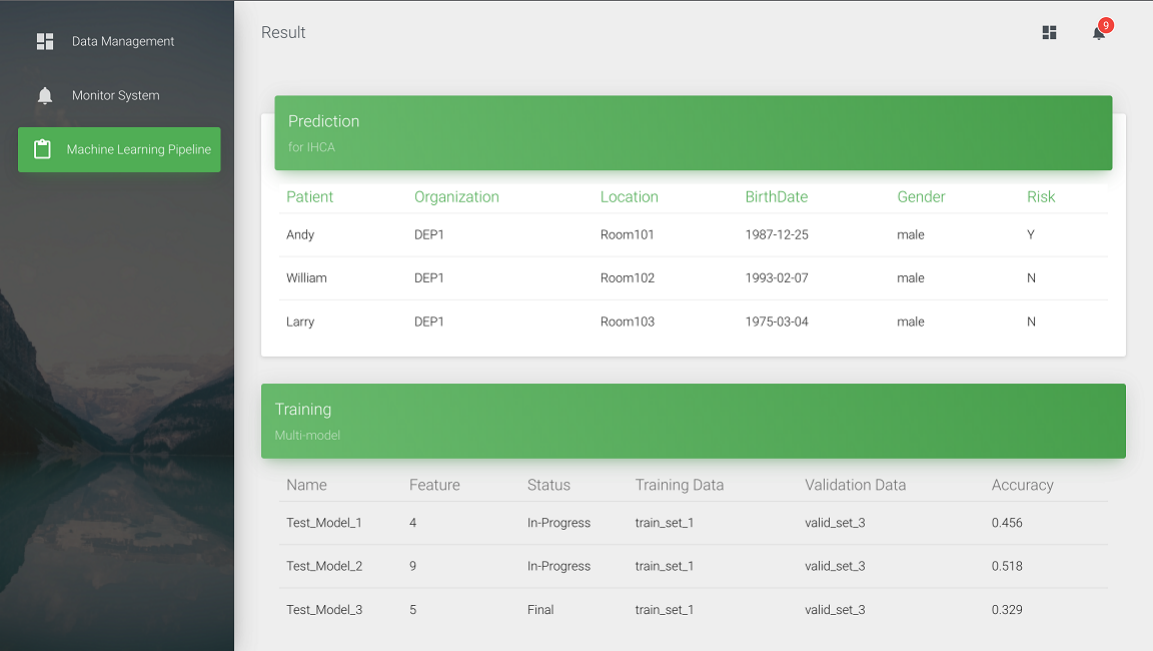
Screenshot of the machine learning pipeline including prediction and training. DEP: department; IHCA: in-hospital cardiac arrest.

**Figure 9:**
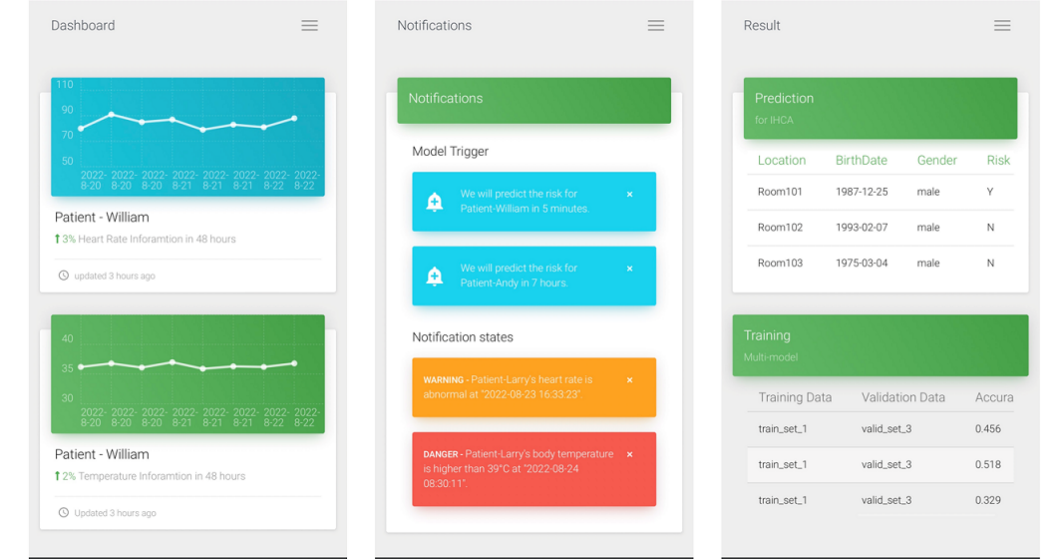
User interface developed using Responsive Web Design for mobile devices. IHCA: in-hospital cardiac arrest.

We converted the 5 vital signs into FHIR observations in JSON format using FHIR ETL. These vital signs include systolic blood pressure, diastolic blood pressure, heart rate, respiratory rate, and body temperature. For demonstration, we used pseudonymization [22] to protect the patient’s privacy. Furthermore, we divided the proposed system into the following 3 components: data management, an early warning system, and a machine learning pipeline. In terms of data management, as shown in Figure 6, we developed a data static dashboard so that it can be accessed by medical staff using a browser. The dashboard uses the HTTP GET method to obtain both the Patient and Observation resources. Then, the patient’s vital signs over the previous 48 hours are displayed. In the early warning system, medical staff can set the vital signs alert threshold to decide whether to show the alert in the notification list as shown in Figure 7. Then, the machine learning pipeline exports the vital signs data from the Observation resource to the FHIR server. We integrated a long short-term memory network–based model [21] using vital signs data to predict IHCA. It used the time series early warning score, which used heart rate, systolic blood pressure, and respiratory data. When the training process of the prediction model is initiated, the status “in progress” will appear in MongoDB. After model training, the status will be updated to “final,” and the dashboard will show the latest accuracy of the model. The proposed dashboard is shown in Figure 8. However, the system can be used on mobile devices as well as desktop computers. We followed the Responsive Web Design [23] to design a user-friendly mobile interface (Figure 9).

## Discussion

### Principal Findings

In this paper, we piloted the use of an implementation guide that combines IHCA with vital signs, which have been widely adopted in IHCA assessment [4,21] and play an important role in inpatient deterioration detection. Many health care institutions have developed early warning score systems to identify hospitalized patients that are at risk of deterioration, and in recent years, they have begun to incorporate machine learning–based models into this process. To promote system interoperability, we used the FHIR standard to achieve consistent information exchange. We also combined 5 resources (Organization, Location, Practitioner, Patient, and Encounter) to represent the EHR. Then, based on the FHIR vital signs profile, we exported vital signs data to HIS database and defined the IHCA implementation. In addition, we developed the 3 FHIR applications of data management using a dashboard, a real-time early warning system, and a machine learning–based pipeline. According to the IHCA implementation guide, our proposed system makes it easy to integrate vital signs–related applications.

### Limitations

The implementation guide was only developed for vital signs–related studies. However, some case studies still need to include treatment history [24], blood urea nitrogen [25], and creatinine [25]. These further improvements can be made to the EHR.

### Comparison With Prior Work

Despite the result that indicated that FHIR can improve the interoperability of health care information systems [26-28], existing studies have only developed the resource and profiles. Seong et al [16] demonstrated how quality information regarding clinical next-generation sequencing genomic testing can be exchanged in a standardized format by profiling an FHIR genomic resource and developing an FHIR-based web application that exchanges quality information. Based on the human-centered design methodology, Park et al [17] developed a worker-centered personal health record (PHR) app for occupational health. The PHRs were managed through a cloud server using Azure API for FHIR, and the PHR FHIR resources included Patient, Organization, DiagnosticReport, Observation, Practitioner, Condition, Procedure, MedicationStatement, Medication, and Encounter. In addition, Chukwu et al [18] profiled FHIR resources for maternal and child health referral use cases. Our study is distinguished from these previous works because we provided the IHCA implementation guidance regarding the use of FHIR resources as a conduit for the data required by the early monitoring system and machine learning. We also proposed a minimum requirements data model and combined it with the FHIR standard. To integrate the early monitoring system and machine learning, we based them on the FHIR vital sign profile and many FHIR resources to extend the data model. Besides, the related studies focus on new application development. In this study, we focus on legacy system integration, so we transfer and synchronize data through FHIR ETL.

### Conclusions

We successfully demonstrated a process that standardizes health care information for inpatient deterioration detection using vital signs. Based on the FHIR definition, we provided an implementation guide that includes data mapping, an integration process, and IHCA assessment using vital signs. We also provided a clarified system architecture that can be used to develop clinical decision support systems. Based on FHIR, we integrated the 3 different systems into 1 dashboard system, which can effectively solve the complexity of the system in the medical staff workflow.

## Appendix

Multimedia Appendix 1 All Health Level 7 Fast Healthcare Interoperability Resources Bundle responses in this study.

## Abbreviations

API: application programming interface
EHR: electronic health record
ETL: Extract Transform Load
FHIR: Fast Healthcare Interoperability Resources
HIS: healthcare information system
IHCA: in-hospital cardiac arrest
PHR: personal health record
