# Supplementary material for: Fast Healthcare Interoperability Resources for Inpatient Deterioration Detection With Time-Series Vital Signs: Design and Implementation Study
Source: JMIR Med Inform. 2022 Oct 13;10(10):e42429. doi: 10.2196/42429 (PMC9614630; doi:10.2196/42429)
Supplement: Multimedia Appendix 1 [file medinform_v10i10e42429_app1.docx]

[/fhir/Patient?_format=json&_pretty=true](http://rd-cicd-pc:8090/fhir/Patient?_format=json&_pretty=true)

{
 "resourceType": "Bundle",
 "id": "6d4700ed-54be-4636-a829-b9e3288082d2",
 "meta": {
 "lastUpdated": "2022-08-23T08:48:38.209+00:00"
 },
 "type": "searchset",
 "total": 3,
 "link": [ {
 "relation": "self",
 "url": "<http://localhost:8080/fhir/Patient?_pretty=true>"
 } ],
 "entry": [ {
 "fullUrl": "<http://localhost:8080/fhir/Patient/1>",
 "resource": {
 "resourceType": "Patient",
 "id": "1",
 "meta": {
 "versionId": "1",
 "lastUpdated": "2022-08-23T06:44:54.769+00:00",
 "source": "#cX6P7so4NiQA6KvY"
 },
 "text": {
 "status": "generated",
 "div": "<div xmlns=\"http://www.w3.org/1999/xhtml\"><div class=\"hapiHeaderText\"/><table class=\"hapiPropertyTable\"><tbody><tr><td>Identifier</td><td>A134587990</td></tr><tr><td>Date of birth</td><td><span>25 December 1987</span></td></tr></tbody></table></div>"
 },
 "identifier": [ {
 "value": "A134587990"
 } ],
 "name": [ {
 "text": "Andy"
 } ],
 "gender": "male",
 "birthDate": "1987-12-25"
 },
 "search": {
 "mode": "match"
 }
 }, {
 "fullUrl": "<http://localhost:8080/fhir/Patient/2>",
 "resource": {
 "resourceType": "Patient",
 "id": "2",
 "meta": {
 "versionId": "1",
 "lastUpdated": "2022-08-23T06:48:14.557+00:00",
 "source": "#PMuj3vQ8RLTanCD4"
 },
 "text": {
 "status": "generated",
 "div": "<div xmlns=\"http://www.w3.org/1999/xhtml\"><div class=\"hapiHeaderText\"/><table class=\"hapiPropertyTable\"><tbody><tr><td>Identifier</td><td>K123035997</td></tr><tr><td>Date of birth</td><td><span>07 February 1993</span></td></tr></tbody></table></div>"
 },
 "identifier": [ {
 "value": "K123035997"
 } ],
 "name": [ {
 "text": "William"
 } ],
 "gender": "male",
 "birthDate": "1993-02-07"
 },
 "search": {
 "mode": "match"
 }
 }, {
 "fullUrl": "<http://localhost:8080/fhir/Patient/3>",
 "resource": {
 "resourceType": "Patient",
 "id": "3",
 "meta": {
 "versionId": "1",
 "lastUpdated": "2022-08-23T06:48:39.650+00:00",
 "source": "#qNQM2Ln15zXsJyj2"
 },
 "text": {
 "status": "generated",
 "div": "<div xmlns=\"http://www.w3.org/1999/xhtml\"><div class=\"hapiHeaderText\"/><table class=\"hapiPropertyTable\"><tbody><tr><td>Identifier</td><td>M129077857</td></tr><tr><td>Date of birth</td><td><span>04 March 1975</span></td></tr></tbody></table></div>"
 },
 "identifier": [ {
 "value": "M129077857"
 } ],
 "name": [ {
 "text": "Larry"
 } ],
 "gender": "male",
 "birthDate": "1975-03-04"
 },
 "search": {
 "mode": "match"
 }
 } ]
}

[/fhir/Practitioner?_format=json&_pretty=true](http://rd-cicd-pc:8090/fhir/Practitioner?_format=json&_pretty=true)

{
 "resourceType": "Bundle",
 "id": "408cd76b-b87e-4395-a37a-8143cfd0ad49",
 "meta": {
 "lastUpdated": "2022-08-23T08:49:32.028+00:00"
 },
 "type": "searchset",
 "total": 1,
 "link": [ {
 "relation": "self",
 "url": "<http://localhost:8080/fhir/Practitioner?_pretty=true>"
 } ],
 "entry": [ {
 "fullUrl": "<http://localhost:8080/fhir/Practitioner/4>",
 "resource": {
 "resourceType": "Practitioner",
 "id": "4",
 "meta": {
 "versionId": "1",
 "lastUpdated": "2022-08-23T07:22:02.350+00:00",
 "source": "#vZsb8YM0DguqKSFY"
 },
 "identifier": [ {
 "value": "DOC001"
 } ],
 "name": [ {
 "text": "Jason"
 } ]
 },
 "search": {
 "mode": "match"
 }
 } ]
}

[/fhir/Organization?_format=json&_pretty=true](http://rd-cicd-pc:8090/fhir/Organization?_format=json&_pretty=true)

{
 "resourceType": "Bundle",
 "id": "7583ddfb-708d-478c-87f7-280071323f05",
 "meta": {
 "lastUpdated": "2022-08-23T08:50:03.510+00:00"
 },
 "type": "searchset",
 "total": 1,
 "link": [ {
 "relation": "self",
 "url": "<http://localhost:8080/fhir/Organization?_pretty=true>"
 } ],
 "entry": [ {
 "fullUrl": "<http://localhost:8080/fhir/Organization/5>",
 "resource": {
 "resourceType": "Organization",
 "id": "5",
 "meta": {
 "versionId": "1",
 "lastUpdated": "2022-08-23T07:30:03.395+00:00",
 "source": "#2t8kBhe3LIntbNyO"
 },
 "identifier": [ {
 "value": "DEP1"
 } ]
 },
 "search": {
 "mode": "match"
 }
 } ]
}

[/fhir/Location?_format=json&_pretty=true](http://rd-cicd-pc:8090/fhir/Location?_format=json&_pretty=true)

{
 "resourceType": "Bundle",
 "id": "06234363-4910-486f-9a12-0acb3b8c7e8b",
 "meta": {
 "lastUpdated": "2022-08-23T08:50:35.123+00:00"
 },
 "type": "searchset",
 "total": 3,
 "link": [ {
 "relation": "self",
 "url": "<http://localhost:8080/fhir/Location?_pretty=true>"
 } ],
 "entry": [ {
 "fullUrl": "<http://localhost:8080/fhir/Location/6>",
 "resource": {
 "resourceType": "Location",
 "id": "6",
 "meta": {
 "versionId": "1",
 "lastUpdated": "2022-08-23T07:30:58.228+00:00",
 "source": "#0cy4iw1S9Hqt9L13"
 },
 "identifier": [ {
 "value": "Loc1"
 } ]
 },
 "search": {
 "mode": "match"
 }
 }, {
 "fullUrl": "<http://localhost:8080/fhir/Location/7>",
 "resource": {
 "resourceType": "Location",
 "id": "7",
 "meta": {
 "versionId": "1",
 "lastUpdated": "2022-08-23T07:31:13.506+00:00",
 "source": "#LeLMZwne2Zr3VKpv"
 },
 "identifier": [ {
 "value": "Loc2"
 } ]
 },
 "search": {
 "mode": "match"
 }
 }, {
 "fullUrl": "<http://localhost:8080/fhir/Location/8>",
 "resource": {
 "resourceType": "Location",
 "id": "8",
 "meta": {
 "versionId": "1",
 "lastUpdated": "2022-08-23T07:31:26.182+00:00",
 "source": "#5w8uqBcXqKpSbugF"
 },
 "identifier": [ {
 "value": "Loc3"
 } ]
 },
 "search": {
 "mode": "match"
 }
 } ]
}

[/fhir/Encounter?_format=json&_pretty=true](http://rd-cicd-pc:8090/fhir/Encounter?_format=json&_pretty=true)

{
 "resourceType": "Bundle",
 "id": "8a8013c5-23d2-4012-b10b-60d041516079",
 "meta": {
 "lastUpdated": "2022-08-23T08:51:27.996+00:00"
 },
 "type": "searchset",
 "total": 3,
 "link": [ {
 "relation": "self",
 "url": "<http://localhost:8080/fhir/Encounter?_pretty=true>"
 } ],
 "entry": [ {
 "fullUrl": "<http://localhost:8080/fhir/Encounter/9>",
 "resource": {
 "resourceType": "Encounter",
 "id": "9",
 "meta": {
 "versionId": "1",
 "lastUpdated": "2022-08-23T08:04:20.014+00:00",
 "source": "#ebqI8zbmemd7zHGX"
 },
 "identifier": [ {
 "value": "vs001"
 } ],
 "status": "in-progress",
 "class": {
 "system": "<http://terminology.hl7.org/CodeSystem/v3-ActCode>",
 "code": "IMP",
 "display": "inpatient encounter"
 },
 "subject": {
 "reference": "Patient/1"
 },
 "participant": [ {
 "individual": {
 "reference": "Practitioner/4"
 }
 } ],
 "location": [ {
 "location": {
 "reference": "Location/6"
 }
 } ],
 "serviceProvider": {
 "reference": "Organization/5"
 }
 },
 "search": {
 "mode": "match"
 }
 }, {
 "fullUrl": "<http://localhost:8080/fhir/Encounter/10>",
 "resource": {
 "resourceType": "Encounter",
 "id": "10",
 "meta": {
 "versionId": "1",
 "lastUpdated": "2022-08-23T08:05:37.480+00:00",
 "source": "#5LiiI3o79hg9LSmU"
 },
 "identifier": [ {
 "value": "vs002"
 } ],
 "status": "in-progress",
 "class": {
 "system": "<http://terminology.hl7.org/CodeSystem/v3-ActCode>",
 "code": "IMP",
 "display": "inpatient encounter"
 },
 "subject": {
 "reference": "Patient/2"
 },
 "participant": [ {
 "individual": {
 "reference": "Practitioner/4"
 }
 } ],
 "location": [ {
 "location": {
 "reference": "Location/7"
 }
 } ],
 "serviceProvider": {
 "reference": "Organization/5"
 }
 },
 "search": {
 "mode": "match"
 }
 }, {
 "fullUrl": "<http://localhost:8080/fhir/Encounter/11>",
 "resource": {
 "resourceType": "Encounter",
 "id": "11",
 "meta": {
 "versionId": "1",
 "lastUpdated": "2022-08-23T08:07:00.328+00:00",
 "source": "#AKctDPTONd6v1E7T"
 },
 "identifier": [ {
 "value": "vs003"
 } ],
 "status": "in-progress",
 "class": {
 "system": "<http://terminology.hl7.org/CodeSystem/v3-ActCode>",
 "code": "IMP",
 "display": "inpatient encounter"
 },
 "subject": {
 "reference": "Patient/3"
 },
 "participant": [ {
 "individual": {
 "reference": "Practitioner/4"
 }
 } ],
 "location": [ {
 "location": {
 "reference": "Location/8"
 }
 } ],
 "serviceProvider": {
 "reference": "Organization/5"
 }
 },
 "search": {
 "mode": "match"
 }
 } ]
}

[/fhir/Observation?_pretty=true](http://localhost:8080/fhir/Observation?_pretty=true)

*{
 "resourceType": "Bundle",
 "id": "324d3de0-c91a-4418-947f-e8d0a7b486c8",
 "meta": {
 "lastUpdated": "2022-08-24T02:01:22.669+00:00"
 },
 "type": "searchset",
 "total": 4,
 "link": [ {
 "relation": "self",
 "url": "*[*http://localhost:8080/fhir/Observation?_pretty=true*](http://localhost:8080/fhir/Observation?_pretty=true)*"
 } ],
 "entry": [ {
 "fullUrl": "*[*http://localhost:8080/fhir/Observation/12*](http://localhost:8080/fhir/Observation/12)*",
 "resource": {
 "resourceType": "Observation",
 "id": "12",
 "meta": {
 "versionId": "1",
 "lastUpdated": "2022-08-23T16:28:48.889+00:00",
 "source": "#MhSJcAXurWLAmcFy",
 "profile": [ "*[*http://hl7.org/fhir/StructureDefinition/vitalsigns*](http://hl7.org/fhir/StructureDefinition/vitalsigns)*" ]
 },
 "status": "final",
 "category": [ {
 "coding": [ {
 "system": "*[*http://terminology.hl7.org/CodeSystem/observation-category*](http://terminology.hl7.org/CodeSystem/observation-category)*",
 "code": "vital-signs",
 "display": "Vital Signs"
 } ],
 "text": "Vital Signs"
 } ],
 "code": {
 "coding": [ {
 "system": "*[*http://loinc.org*](http://loinc.org)*",
 "code": "8867-4",
 "display": "Heart rate"
 } ],
 "text": "Heart rate"
 },
 "subject": {
 "reference": "Patient/1"
 },
 "effectiveDateTime": "2022-08-01T13:00:00-08:00",
 "valueQuantity": {
 "value": 69,
 "unit": "beats/minute",
 "system": "*[*http://unitsofmeasure.org*](http://unitsofmeasure.org)*",
 "code": "/min"
 }
 },
 "search": {
 "mode": "match"
 }
 }, {
 "fullUrl": "*[*http://localhost:8080/fhir/Observation/16*](http://localhost:8080/fhir/Observation/16)*",
 "resource": {
 "resourceType": "Observation",
 "id": "16",
 "meta": {
 "versionId": "1",
 "lastUpdated": "2022-08-23T16:33:23.023+00:00",
 "source": "#Am96GYyTbB9vX3fm",
 "profile": [ "*[*http://hl7.org/fhir/StructureDefinition/vitalsigns*](http://hl7.org/fhir/StructureDefinition/vitalsigns)*" ]
 },
 "status": "final",
 "category": [ {
 "coding": [ {
 "system": "*[*http://terminology.hl7.org/CodeSystem/observation-category*](http://terminology.hl7.org/CodeSystem/observation-category)*",
 "code": "vital-signs",
 "display": "Vital Signs"
 } ],
 "text": "Vital Signs"
 } ],
 "code": {
 "coding": [ {
 "system": "*[*http://loinc.org*](http://loinc.org)*",
 "code": "8310-5",
 "display": "Body temperature"
 } ],
 "text": "Body temperature"
 },
 "subject": {
 "reference": "Patient/1"
 },
 "effectiveDateTime": "2022-08-01T13:00:00-08:00",
 "valueQuantity": {
 "value": 36.9,
 "unit": "C",
 "system": "*[*http://unitsofmeasure.org*](http://unitsofmeasure.org)*",
 "code": "Cel"
 }
 },
 "search": {
 "mode": "match"
 }
 }, {
 "fullUrl": "*[*http://localhost:8080/fhir/Observation/17*](http://localhost:8080/fhir/Observation/17)*",
 "resource": {
 "resourceType": "Observation",
 "id": "17",
 "meta": {
 "versionId": "1",
 "lastUpdated": "2022-08-23T16:34:01.766+00:00",
 "source": "#QB41XmTTaYPHpccr",
 "profile": [ "*[*http://hl7.org/fhir/StructureDefinition/vitalsigns*](http://hl7.org/fhir/StructureDefinition/vitalsigns)*" ]
 },
 "status": "final",
 "category": [ {
 "coding": [ {
 "system": "*[*http://terminology.hl7.org/CodeSystem/observation-category*](http://terminology.hl7.org/CodeSystem/observation-category)*",
 "code": "vital-signs",
 "display": "Vital Signs"
 } ],
 "text": "Vital Signs"
 } ],
 "code": {
 "coding": [ {
 "system": "*[*http://loinc.org*](http://loinc.org)*",
 "code": "9279-1",
 "display": "Respiratory rate"
 } ],
 "text": "Respiratory rate"
 },
 "subject": {
 "reference": "Patient/1"
 },
 "effectiveDateTime": "2022-08-01T13:00:00-08:00",
 "valueQuantity": {
 "value": 29,
 "unit": "breaths/minute",
 "system": "*[*http://unitsofmeasure.org*](http://unitsofmeasure.org)*",
 "code": "/min"
 }
 },
 "search": {
 "mode": "match"
 }
 }, {
 "fullUrl": "*[*http://localhost:8080/fhir/Observation/18*](http://localhost:8080/fhir/Observation/18)*",
 "resource": {
 "resourceType": "Observation",
 "id": "18",
 "meta": {
 "versionId": "1",
 "lastUpdated": "2022-08-23T16:34:23.274+00:00",
 "source": "#074yuBCRO0QdBmba",
 "profile": [ "*[*http://hl7.org/fhir/StructureDefinition/vitalsigns*](http://hl7.org/fhir/StructureDefinition/vitalsigns)*" ]
 },
 "status": "final",
 "category": [ {
 "coding": [ {
 "system": "*[*http://terminology.hl7.org/CodeSystem/observation-category*](http://terminology.hl7.org/CodeSystem/observation-category)*",
 "code": "vital-signs",
 "display": "Vital Signs"
 } ]
 } ],
 "code": {
 "coding": [ {
 "system": "*[*http://loinc.org*](http://loinc.org)*",
 "code": "85354-9",
 "display": "Blood pressure panel with all children optional"
 } ],
 "text": "Blood pressure systolic & diastolic"
 },
 "subject": {
 "reference": "Patient/1"
 },
 "effectiveDateTime": "2022-08-01T13:00:00-08:00",
 "performer": [ {
 "reference": "Practitioner/4"
 } ],
 "interpretation": [ {
 "coding": [ {
 "system": "*[*http://terminology.hl7.org/CodeSystem/v3-ObservationInterpretation*](http://terminology.hl7.org/CodeSystem/v3-ObservationInterpretation)*",
 "code": "L",
 "display": "low"
 } ],
 "text": "Below low normal"
 } ],
 "bodySite": {
 "coding": [ {
 "system": "*[*http://snomed.info/sct*](http://snomed.info/sct)*",
 "code": "368209003",
 "display": "Right arm"
 } ]
 },
 "component": [ {
 "code": {
 "coding": [ {
 "system": "*[*http://loinc.org*](http://loinc.org)*",
 "code": "8480-6",
 "display": "Systolic blood pressure"
 }, {
 "system": "*[*http://snomed.info/sct*](http://snomed.info/sct)*",
 "code": "271649006",
 "display": "Systolic blood pressure"
 }, {
 "system": "*[*http://acme.org/devices/clinical-codes*](http://acme.org/devices/clinical-codes)*",
 "code": "bp-s",
 "display": "Systolic Blood pressure"
 } ]
 },
 "valueQuantity": {
 "value": 118,
 "unit": "mmHg",
 "system": "*[*http://unitsofmeasure.org*](http://unitsofmeasure.org)*",
 "code": "mm[Hg]"
 },
 "interpretation": [ {
 "coding": [ {
 "system": "*[*http://terminology.hl7.org/CodeSystem/v3-ObservationInterpretation*](http://terminology.hl7.org/CodeSystem/v3-ObservationInterpretation)*",
 "code": "N",
 "display": "normal"
 } ],
 "text": "Normal"
 } ]
 }, {
 "code": {
 "coding": [ {
 "system": "*[*http://loinc.org*](http://loinc.org)*",
 "code": "8462-4",
 "display": "Diastolic blood pressure"
 } ]
 },
 "valueQuantity": {
 "value": 55,
 "unit": "mmHg",
 "system": "*[*http://unitsofmeasure.org*](http://unitsofmeasure.org)*",
 "code": "mm[Hg]"
 },
 "interpretation": [ {
 "coding": [ {
 "system": "*[*http://terminology.hl7.org/CodeSystem/v3-ObservationInterpretation*](http://terminology.hl7.org/CodeSystem/v3-ObservationInterpretation)*",
 "code": "L",
 "display": "low"
 } ],
 "text": "Below low normal"
 } ]
 } ]
 },
 "search": {
 "mode": "match"
 }
 } ]
}*
